# Sulfur Use Efficiency Is a Significant Determinant of Drought Stress Tolerance in Relation to Photosynthetic Activity in *Brassica napus* Cultivars

**DOI:** 10.3389/fpls.2016.00459

**Published:** 2016-04-08

**Authors:** Bok-Rye Lee, Rashed Zaman, Jean-Christophe Avice, Alain Ourry, Tae-Hwan Kim

**Affiliations:** ^1^Department of Animal Science, Institute of Agricultural Science and Technology, College of Agriculture and Life Science, Chonnam National UniversityGwangju, South Korea; ^2^Biotechnology Research Institute, Chonnam National UniversityGwangju, South Korea; ^3^Université de Caen Basse-NormandieCaen, France; ^4^UMR INRA-UCBN 950 Ecophysiologie Végétale, Agronomie et Nutritions N, Université de Caen Basse NormandieCaen, France

**Keywords:** *Brassica napus*, PEG-induced drought stress, photosynthetic activity, ^34^S tracing, sulfur use efficiency

## Abstract

To investigate the varietal difference in sulfur use efficiency (SUE) and drought stress tolerance, *Brassica napus* ‘Mosa’ and ‘Saturnin’ were exposed to polyethylene glycol (PEG)-induced drought stress for 72 h. Direct quantification of S uptake, *de novo* synthesis of amino acids and proteins was performed by tracing ^34^S. The responses of photosynthetic activity in relation to SUE were also examined. The total amount of newly absorbed S decreased with drought stress in both cultivars but the decrease rate was significantly higher in Mosa (-64%) than in Saturnin (-41%). Drought stress also decreased the amount of S assimilated into amino acids (^34^S-amino acids) and proteins (^34^S-proteins). The total amount of S incorporated into amino acids and proteins was generally higher in Saturnin (663.7 μg S per plant) than in Mosa (337.3 μg S per plant). The estimation of SUE based on S uptake (SUpE) and S assimilation (SUaE) showed that SUE was much higher in Saturnin than in Mosa. The inhibition of photosynthetic activity including Rubisco protein degradation caused by drought stress was much lower in the cultivar with higher SUE (Saturnin). The present study clearly indicates that the genotype with higher SUE is more tolerant to PEG-induced drought stress.

## Introduction

Oilseed rape (*Brassica napus* L.) is increasingly grown throughout the world. It has a wide range of uses including vegetable oil, animal feed, and alternative fuel (Abdallah et al., 2010). Oilseed rape is also considered to be an excellent rotation crop which enhances suppression of soil-borne pathogens and a catch crop species that limits leaching of mineral nutrients into the aquifer (Kirkegaard et al., 1997). World production of oilseed rape is growing rapidly, with FAO reporting that 47 million tons of oilseed rape was produced in 2007, and 58.4 million tons estimated to have been produced in the 2010–2011 (Saeidnia and Gohari, 2012).

In general, oilseed rape, and *Brassica* species have a characteristically high sulfur (S) demand during vegetative growth for the synthesis of proteins. For example, the production of 1 ton of rape seeds requires about 16 kg S (Blake-Kalff et al., 2001), compared with 2–3 kg of S per ton of wheat grain (Zhao et al., 1999). Moreover, these plants contain relatively high amounts of S-containing secondary metabolites, viz. glucosinolate, which accounts for up to 20% of the organic sulfur content (Castro et al., 2004; Aghajanzadeh et al., 2014). However, S-deficiency is a common phenomenon in many agro-ecosystems, caused by a massive decrease of S inputs from atmospheric deposition and reduced application of S-containing fertilizer in the last three decades (Zhao et al., 1997). S-deficiency and/or S-deprivation decrease the cell sap osmotic potential due to a net increase of intracellular solutes, rather than from a loss of cell water (Kusaka et al., 2005). In addition, S-deficiency reduces chlorophyll and Rubisco content, and provokes chlorosis of young leaves (Gilbert et al., 1997; Lee et al., 2014; Muneer et al., 2014). These results imply that S-deficiency results in a general inhibition of photosynthesis and protein synthesis. In *B. napus*, studies carried out under controlled greenhouse (Balint and Rengel, 2008; Dubousset et al., 2010) or field conditions (Schnug et al., 1993; Fismes et al., 2000) have shown that S-deficiency reduces N-use efficiency, and that N-deficiency can also affect the S-use efficiency. Under S-deficiency conditions, a reduction in the internal S pools and an increase in soluble nitrogen content, including nitrate and amides, have been observed (Prosser et al., 1997; Warrilow and Hawkesford, 1998). In *B. napus*, our recent work with ^15^N and ^34^S tracing revealed that the total amount of N and S incorporated into proteins decreased by 28.8 and 62.1% under S-deficiency conditions (Lee et al., 2013). Our work also showed significant modification of the partitioning of newly absorbed N and S, and remobilization of stored N and S, in S-deficient plants (Lee et al., 2014).

Several studies have indicated that S nutrition plays a role in stress tolerance and defense mechanisms. For examples, thiol-containing compounds, especially reduced GSH, which is sensitive to oxidized environments, may be modulators of the stress response (Khan et al., 2009; Szalai et al., 2009). GSH has been shown to take part in the removal of reactive oxygen species (Rausch et al., 2007; Astolfi and Zuchi, 2013). Sulfur is an essential element in the formation of sulfhydryl (S-H) and disulfide bonds (S-S). These bonds are important for the stabilization of protein structures (Saito, 2000). In this context, the role of S nutrition in alleviating negative responses to salinity stress (Khan et al., 2009; Astolfi and Zuchi, 2013; Fatma et al., 2014) and iron deficiency (Zuchi et al., 2009; Muneer et al., 2014) have been widely reported.

Drought stress is one limiting factor to plant growth. In Korea, drought occurs predominantly from March to the early June, when the growth and development of most varieties of oilseed rape (winter type) actively progress. Oilseed rape is, therefore, often exposed to drought stress during the early stages of growth. Selection for physiologically efficient S-use cultivars may have value in breeding programs aimed at improving stress tolerance, grain yield, and quality. In this study, we hypothesized that cultivar variation in SUE under PEG-induced drought stress may be attributed to two components: (i) S-uptake efficiency (SUpE; S uptake per S supplied) and (ii) S-assimilation efficiency (SUaE; S assimilated to amino acids and proteins per S supplied), and that the genotype producing higher SUE is more tolerant to PEG-induced drought stress. To test this hypothesis, S uptake and the amount of S incorporated into amino acids and proteins were directly measured by a ^34^S tracing method. The responses to PEG-induced drought stress of parameters related to photosynthetic activity were also assessed in relation to SUE in two *B. napus* cultivars.

## Materials and Methods

### Plant Culture

The surface-sterilized seeds of *B. napus* ‘Mosa’ and ‘Saturnin’ were sown into bed soil in a tray. At the three-leaf stage, seedlings were transferred to 2.5 L pots filled with hydroponic nutrient solution containing (mM for the macro elements): 1.0 NH_4_NO_3_; 0.4 KH_2_PO_4_; 1.0 K_2_SO_4_; 0.5 K_2_HPO_4_; 3.0 CaCl_2_; 0.5 MgSO_4_; 0.15 K_2_HPO_4_; 0.2 Fe-Na EDTA; and (μM for the micro elements): 14 H_3_BO_3_; 5.0 MnSO_4_·H_2_O, 3.0 ZnSO_4_·7H_2_O; 0.7 CuSO_4_·5H_2_O; 0.7 (NH_4_)_6_Mo_7_O_24_; 0.1 CoCl_2._ Seedlings were then grown in a greenhouse. The nutrient solution was continuously aerated and renewed every 5 days. Natural light was supplemented by metal halide lamps, which generated *c*. 400 μmol photons m^-2^ s^-1^ at the canopy height for 16 h per day.

### PEG-Induced Drought Stress and Isotope Labeling

Eight-week-old plants were divided in two groups for the application of (PEG-6000). One group of experimental plants was supplied normal nutrient solution as a control. The other group was supplied 8% PEG-6000 with normal nutrient solution for 72 h. For the ^34^S feeding, S sources in hydroponic solution were replaced by ^34^S labeling solution containing 1.5 mM K_2_^34^SO_4_ with 1.0% ^34^S atom excess.

### Measurements and Sampling

Leaf water potential was measured as the petiole xylem-pressure potential using a pressure chamber (PMS Instrument Co. Corvallis, OR, USA). Photosynthesis rate, stomatal conductance and transpiration were measured in a greenhouse using a portable photosynthesis measurement system (LI-6400. LI-COR, Inc. Lincoln, NE, USA). One fully expanded mature leaf per plant was tagged with a small wire, and measurements were taken over 72 h of treatment. Measurement was done every day, 4 h after the beginning of the photoperiod on the same tagged leaf. The first sampling (0 h) was conducted just before the drought stress treatment (the beginning of ^34^SO_4_^2-^ feeding) was applied at 10:00 h. Additional measurements were taken at 24, 48, and 72 h after treatment. The harvested plants were separated into leaves and roots. All plant samples were immediately frozen in liquid nitrogen after harvest, and then freeze-dried, weighed, ground, and stored in vacuum desiccators for further analysis.

### Chemical Fractionation and Isotope Analysis

About 200 mg of finely ground freeze-dried samples were extracted twice with 100 mM sodium-phosphate buffer (pH 7.5) at 4°C. Proteins in the combined supernatant were precipitated with 80% (v/v) acetone and centrifuged at 10,000× *g* at 4°C for 10 min. The resulting pellets, which corresponded to NAS incorporated into protein fractions (^34^S-protein), were re-suspended in 0.5 mL of ultra-pure water. To measure NAS in sulfate (^34^S-sulfate), the aliquot obtained after protein precipitation was evaporated under vacuum at 4°C and centrifuged. The resulting supernatant was passed through an H^+^ column (Dowex 50W × 8). The pH of the solution collected from the H^+^ column was adjusted to neutral pH, and this solution was concentrated to a final volume of 0.5 mL. The NAS incorporated into amino acids (^34^S-amino acids) was eluted from the Dowex 50W × 8 column by using 25 mL of 0.5 M HCl and concentrated to 1 mL. For the fractionated liquid samples, an appropriate sample volume (usually 0.1 mL) was dropped into a tin capsule and freeze-dried before S and ^34^S quantification. The NAS (^34^S-total S) was obtained from 5 mg of freeze-dried powder samples.

The S content and ^34^S atom % of all fractions was determined by a continuous flow isotope mass spectrometer (IsoPrime, GV Instrument, Manchester, UK). The ^34^S abundances measured were converted to (RSA, i.e., % of recently incorporated atoms relative to the total numbers of atoms in the sample) via the following equation (1):

(1) RSA=(S34⁢ atom⁢ %  measured−natural⁢ S34⁢ atom⁢  %)/  (S34⁢ atom⁢ % of⁢ fed⁢ SO42−−natural⁢ S3434atom⁢ %)×100⁢

in which the natural ^34^S atom % was adopted from the ^34^S atom % of non-^34^S -fed plants.

The amounts of NAS in the S containing compounds were calculated using equation (2):

(2) NAS=(RSA×S⁢  content⁢ ⁢measured⁢ in⁢ a⁢ compound)/100.⁢

### Determination of Sulfur Use Efficiency

Sulfur use efficiency was assessed based on S uptake and S assimilation, respectively, in controls and PEG-induced drought stressed plants. SUE based on S uptake (SUpE) was calculated as the total amount of NAS (^34^S) per gram of S provided in the nutrient solution during 72 h of the experiment, and expressed by mg S taken up g^-1^ S fed. SUE based on S assimilation (SUaE) was calculated as the total amount of S assimilated into amino acids and proteins (^34^S-amino acid + ^34^S-protein) per gram of S provided in the nutrient solution during 72 h of the experiment, and expressed by mg S assimilated g^-1^ S fed.

### SDS-PAGE and Quantification of Rubisco Protein

Leaf samples were homogenized in chilled mortar with ice-cold extraction buffer containing 25 mM Tris-HCl (pH 7.8), 50 mM MgCl_2_, 2.5 mM EDTA, and 1 mM DTT. The homogenate was centrifuged at 10,000× *g* at 4°C for 10 min and the supernatant was applied to SDS polyacrylamide gel electrophoresis (SDS-PAGE) for determination of Rubisco content. Twenty microgram proteins were separated in 1.0 mm thick gels containing 12.5% acrylamide (propenamide). The large subunit of Rubisco was detected by staining with Coomassie brilliant blue R-250. The individual band was extracted with formamide, and quantified by the Bradford dye binding assay using BSA as a standard (Makino et al., 1985).

### Statistical Analysis

Analysis of variance (ANOVA) and Duncan’s multiple range test were employed to compare the means of each treatment. Statistical significance was postulated at *P* < 0.05. Statistical analysis of physiological and biochemical measurements was carried out using the software SAS 9.1.3.

## Results

### PEG-Induced Drought Stress Effects on Leaf Water Potential and Biomass in *B. napus* Cultivars

Varietal differences and PEG-induced drought stress effects on Ψ_w_ were significant (**Table 1**). Drought stress decreased the Ψ_w_ gradually in both cultivars throughout the experiment, showing higher decrease in Mosa (-66.7% at 72 h after treatment) than in Saturnin (-56.9%) (**Table 2**). The Ψ_w_ decreased in drought-stressed leaves, accompanied by the decrease in chlorophyll content in both cultivars. Varietal differences in biomass of drought-stressed plants was significance only in leaves (**Table 1**), and was overall higher in Saturnin in both controls and drought-stressed plants. A significant decrease in leaf biomass, caused by drought-stress, was observed only in Mosa at 72 h after treatment (**Table 2**).

**Table 1:** Analysis of variance (ANOVA) for physiological parameters, sulfate metabolites, SUE based on S-uptake (SUpE), and its assimilation (SUaE) as affected by PEG-induced drought stress in two *Brassica napus* cultivars.

ANOVA	df	Ψ_w_ (MPa)	Chl. (SPAD)	Biomass		NAS		^34^S-sulfate		^34^S -amino acids		^34^S –protein		SUpE	SUaE
				Leaves	Root	Leaves	Root	Leaves	Root	Leaves	Root	Leaves	Root		
Cultivar (C)	1	*	*	***	NS	***	*	***	NS	***	***	***	***	***	***
Stress (S)	1	***	***	**	NS	***	*	**	*	***	***	***	**	***	***
CxS	1	*	NS	NS	NS	NS	NS	NS	NS	NS	NS	NS	NS	**	NS

Asterisks indicate significantly different; *P < 0.05, **P < 0.01, ***P < 0.001; NS, no significance; Chl., Chlorophyll; NAS, newly absorbed S.

**Table 2:** Changes in Ψ_w_, total chlorophyll and biomass of *B. napus* ‘Mosa’ and ‘Saturnin’ under control or PEG-induced drought stress condition for 72 h.

Physiological parameters/Treatment		Hours after treatment			
		0	24	48	72
**Leaf water potential *(Ψ_w_*, MPa)**					
Mosa	control	-0.41 ± 0.04a	-0.42 ± 0.05a	-0.42 ± 0.05a	-0.42 ± 0.04a
	PEG-stressed		-0.70 ± 0.09b	-0.95 ± 0.10b	-1.26 ± 0.09c
Saturnin	control	-0.45 ± 0.07a	-0.44 ± 0.06a	-0.42 ± 0.03a	-0.44 ± 0.06a
	PEG-stressed		-0.68 ± 0.13b	-0.86 ± 0.08b	-1.02 ± 0.11b
**Total chlorophyll content (mg g^-1^ FW)**					
Mosa	control	2.85 ± 0.07b	3.15 ± 0.28ab	3.17 ± 0.61a	2.99 ± 0.39a
	PEG-stressed		2.55 ± 0.32c	1.44 ± 0.11b	0.88 ± 0.14c
Saturnin	control	3.28 ± 0.17a	3.28 ± 0.12a	3.47 ± 0.04a	3.38 ± 0.31a
	PEG-stressed		2.71 ± 0.04bc	1.92 ± 0.09b	1.36 ± 0.07b
**Biomass (g plant^-1^)**					
Leaves					
Mosa	control	9.78 ± 0.65b	9.32 ± 0.21b	9.44 ± 0.34bc	9.43 ± 0.41b
	PEG-stressed		9.21 ± 0.56b	8.57 ± 0.21c	7.56 ± 0.38c
Saturnin	control	11.87 ± 0.33a	11.78 ± 0.54a	11.84 ± 1.12a	12.17 ± 0.57a
	PEG-stressed		11.68 ± 0.95a	10.79 ± 0.81ab	11.00 ± 0.74a
Roots					
Mosa	control	4.79 ± 0.25b	4.82 ± 0.34a	5.28 ± 0.40a	5.05 ± 0.12a
	PEG-stressed		4.75 ± 0.08a	5.25 ± 0.45a	5.45 ± 0.37a
Saturnin	control	5.63 ± 0.36a	5.66 ± 0.45a	5.88 ± 0.57a	5.72 ± 0.52a
	PEG-stressed		5.65 ± 0.48a	5.65 ± 0.51a	5.78 ± 0.42a

*Values are means ± SD of three replicates. Values in a vertical column followed by different letters are significantly different at P < 0.05 according to Duncan’s multiple range test*.

### Amounts of Newly Absorbed S in Total S and in Sulfate Fraction

Cultivar variation in NAS in total S (^34^S-total S) was significant in both leaves and roots (**Table 1**). Sum of ^34^S-total S in leaves and roots (^34^S-total S) at 72 h of treatment was 24.1 mg S plant^-1^ in Mosa and 28.7 mg S plant^-1^ in Saturnin for the non-stressed control plants (**Table 3**). PEG-induced drought stress decreased the amount of ^34^S-total S in leaves and showed a significant cultivar difference, with a higher decrease in Mosa (-80.3%) than in Saturnin (-49.8%). A significant decrease in the amount of ^34^S-total S in roots (-40.6%) was apparent only in Mosa (**Table 3**).

**Table 3:** Changes in amount of NAS (^34^S-total S) and non-assimilated sulfate (^34^S-sulfate) in leaves and roots of *B. napus* ‘Mosa’ and ‘Saturnin’ under control or PEG-induced drought stress condition for 72 h.

Physiological parameters/Treatment			Hours after treatment		
		0	24	48	72
**Newly absorbed S (^34^S-total S, mg plant^-1^)**					
Leaves					
Mosa	control	-	4.74 ± 0.65b	9.80 ± 1.03b	13.99 ± 0.87b
	PEG-stressed		0.98 ± 0.14c	1.89 ± 0.26d	2.75 ± 0.34d
Saturnin	control	-	7.73 ± 0.81a	15.06 ± 0.55a	21.98 ± 0.55a
	PEG-stressed		3.69 ± 0.44b	7.31 ± 0.36c	11.03 ± 0.40c
Roots					
Mosa	control	-	2.94 ± 0.23a	6.85 ± 0.25a	10.14 ± 0.35a
	PEG-stressed		1.88 ± 0.15b	3.78 ± 0.25b	5.96 ± 0.28b
Saturnin	control	-	2.43 ± 0.40ab	4.39 ± 0.68b	6.67 ± 0.57b
	PEG-stressed		1.92 ± 0.15b	3.67 ± 0.34b	5.90 ± 0.53b
**Non-assimilated sulfate (^34^S-sulfate, μg plant^1^)**					
Leaves					
Mosa	control	-	63.8 ± 2.5ab	130.5 ± 2.9bc	200.1 ± 10.5b
	PEG-stressed		57.3 ± 9.8b	105.4 ± 15.8c	142.2 ± 20.6c
Saturnin	control	-	83.2 ± 3.5a	172.5 ± 9.1a	268.6 ± 11.0a
	PEG-stressed		76.4 ± 14.8ab	148.6 ± 16.5ab	221.7 ± 25.2b
Roots					
Mosa	control	-	58.2 ± 4.3a	130.5 ± 20.4bc	195.4 ± 27.9ab
	PEG-stressed		67.1 ± 10.9a	161.5 ± 14.0a	250.0 ± 17.6a
Saturnin	control	-	54.6 ± 6.3a	108.5 ± 14.9c	162.6 ± 20.7b
	PEG-stressed		62.3 ± 12.5a	130.0 ± 23.7bc	202.1 ± 32.0ab

*Values are means ± SD of three replicates. Values in a vertical column followed by different letters are significantly different at P < 0.05 according to Duncan’s multiple range test.*

The amount of NAS in sulfate fraction (^34^S-sulfate) in leaves showed cultivar difference in both controls and drought stressed plants (**Table 3**). PEG-induced drought stress decreased the amount of ^34^S-sulfate in leaves by 28.9 and 17.5%, respectively, in Mosa and Saturnin at 72 h after treatment, when compared to controls. However, PEG-induced drought stress overall increased the amount of ^34^S-sulfate in roots, while cultivar variation was not significant (**Tables 1** and **3**).

### *De novo* Synthesis of Amino Acids and Proteins

**FIGURE 1:**
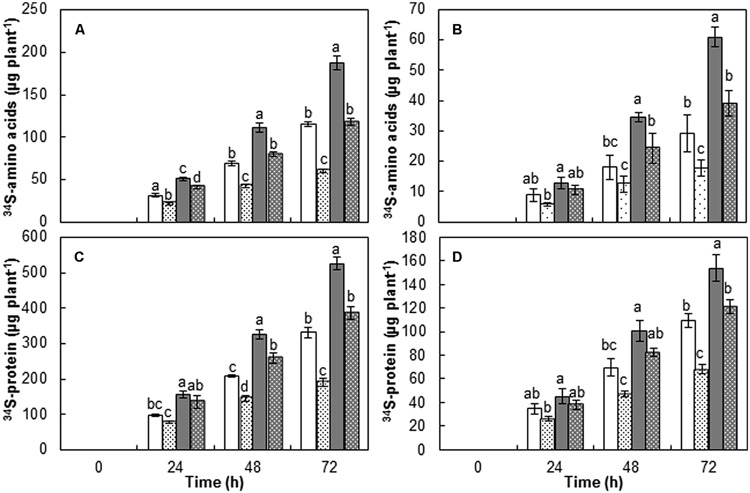
**Amount of S assimilated into amino acids and proteins in leaves (A,C) and roots (B,D) of cultivars Mosa (white bar) or Saturnin (dark gray bar) under control (non-dotted bar) or PEG-induced drought stress (dotted bar) conditions for 72 h.** Data are presented as mean ± SE for *n* = 3. Means denoted by the different letter are significantly different at *P* < 0.05 according to the Duncan’s multiple range test.

To investigate the assimilation of SO_4_^2-^ taken up during 72 h of PEG-induced drought stress, the amount of NAS incorporated into amino acids (^34^S-amino acids) or proteins (^34^S-proteins) were quantified. Cultivar variation and drought stress effects on the ^34^S-amino acids were significant (*P* < 0.001) (**Table 1**). Overall, the amount of ^34^S-amino acids was higher in Saturnin than in Mosa in both controls and drought-stressed plants for the entire course of the experiment (**Figures 1A,B**). PEG-induced drought stress decreased ^34^S-amino acids significantly in both leaves and roots with varietal difference, showing 47.8 and 39.1% decreases in the leaves and roots of Mosa versus 37.0 and 35.7% in Saturnin at 72 h after treatment, when compared to controls (**Figures 1A,B**).

Cultivar variation and drought stress effects for the ^34^S-proteins were also highly significant (*P* < 0.001) (**Table 1**). Overall, the amount of ^34^S-proteins was higher than those assimilated into other fractions, particularly in the leaves (**Figures 1C,D**). Varietal difference in ^34^S-protein was also observed in non-stressed controls (331.4 and 109.1 μg S plant^-1^ in the leaves and roots of Mosa versus 526.0 and 153.9 μg S plant^-1^, respectively, in Saturnin at 72 h after treatment). Drought stress decreased ^34^S-proteins significantly in both leaves and roots. The decreases in ^34^S-proteins caused by drought stress was higher in leaves than in roots with cultivar variation, showing 42.3 and 37.7% decrease in the leaves and roots of Mosa versus 26.8 and 21.2%, respectively, in Saturnin at 72 h after treatment when compared to controls (**Figures 1C,D**).

### Sulfur Use Efficiency Based on S Uptake and Its Assimilation Into Amino Acids and Proteins

**FIGURE 2:**
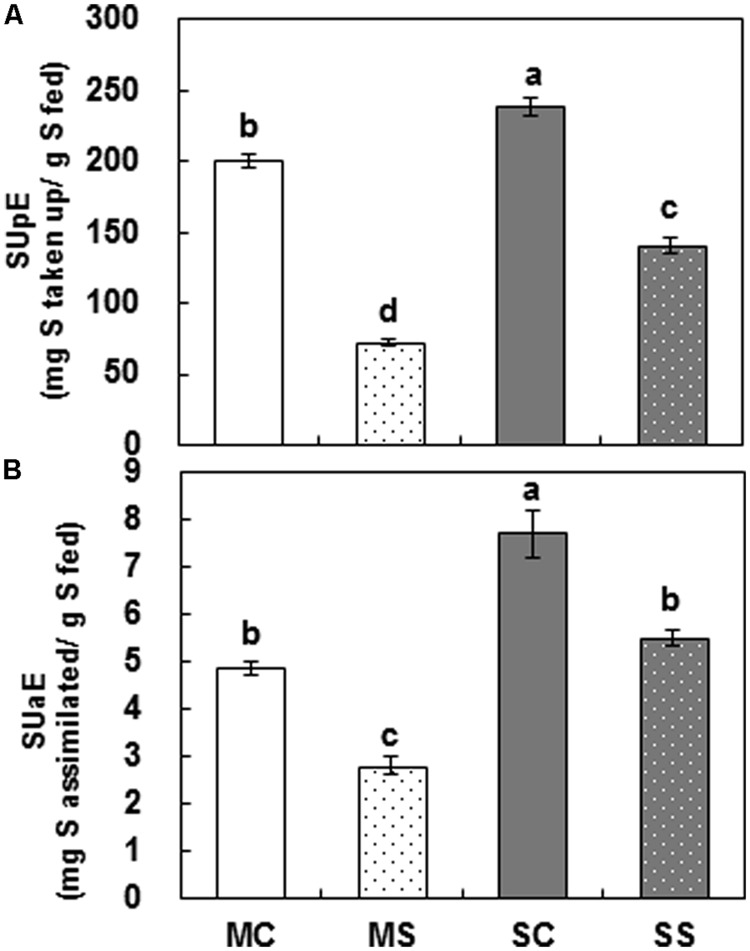
**Sulfur use efficiency based on S uptake (SUpE) (A) and S assimilation (SUaE) (B) of cultivars Mosa (white bar) or Saturnin (dark gray bar) under control (non-dotted bar) or PEG-induced drought stress (dotted bar) conditions at 72 h.** Data are presented as mean ± SE for *n* = 3. Means denoted by the different letter are significantly different at *P* < 0.05 according to the Duncan’s multiple range test.

Cultivar variation in SUE based on S uptake (SUpE), calculated by dividing total NAS by the amount of S supplied for 72 h of treatment, was significant (**Table 1**). SUpE in control plants was 200.4 and 238.0 mg S uptake g^-1^ S fed, respectively, in Mosa and Saturnin (**Figure 2A**). PEG-induced drought stress resulted in a reduction of SUpE with varietal difference, showing higher reduction in Mosa (-66.7%) than in Saturnin (-40.4%).

Sulfur use efficiency based on S assimilation (SUaE), calculated by dividing total amount of S assimilated into amino acids and proteins by the amount of S supplied for a given time, are presented in **Figure 2B**. Cultivar variation was significant (*P* < 0.001) for SUaE, with a range from 4.86 mg S assimilated g^-1^ S taken up (Mosa) to 7.71 mg (Saturnin) in controls and from 2.80 mg S assimilated g^-1^ S taken up (Mosa) to 5.51 mg (Saturnin) in PEG-induced drought stressed plants. The decrease in SUaE due to drought stress was largely higher in Mosa (-42.3%) than in Saturnin (-28.5%).

### Rubisco Protein Content and Photosynthetic Activity

**FIGURE 3:**
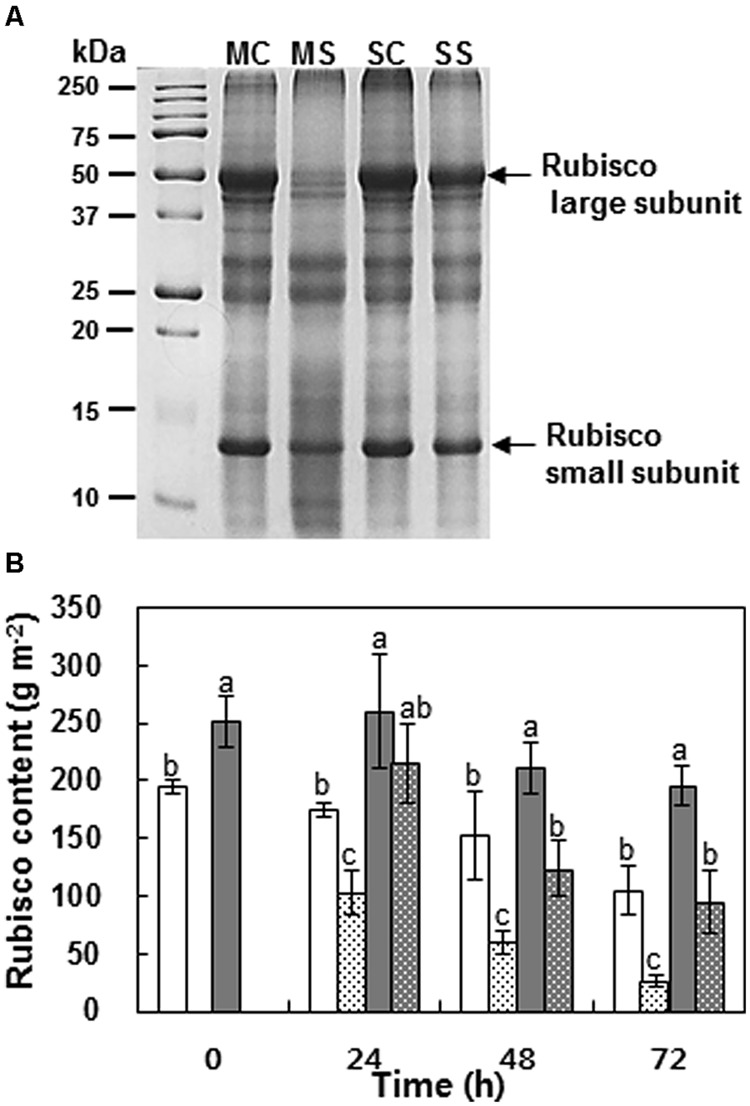
**Leaf protein pattern after SDS–PAGE of cultivars Mosa (M) and Saturnin (S) at 72 h after control (MC or SC) and PEG-induced drought stress (MS or SS) treatment (A).** Changes in Rubisco protein content **(B)** of leaves of Mosa (white bar) or Saturnin (dark gray bar) under control (non-dotted bar) or PEG-induced drought stress (dotted bar) conditions for 72 h. Data are presented as mean ± SE for *n* = 3. Means denoted by the different letter are significantly different at *P* < 0.05 according to the Duncan’s multiple range test.

Rubisco protein patterns showed considerable varietal difference in PEG-induced drought stressed plants (**Figure 3A**). PEG-induced drought stress degraded the prominently large subunits of Rubisco in the low intensity band and slightly degraded the smaller subunits in Mosa (**Figure 3A** Lane 2–3), while much less change was observed in Saturnin (**Figure 3A** Lane 4–5). The decrease in Rubisco protein due to drought stress was estimated as 75.6% in Mosa and 51.1% in Saturnin at 72 h after treatment (**Figure 3B**).

**FIGURE 4:**
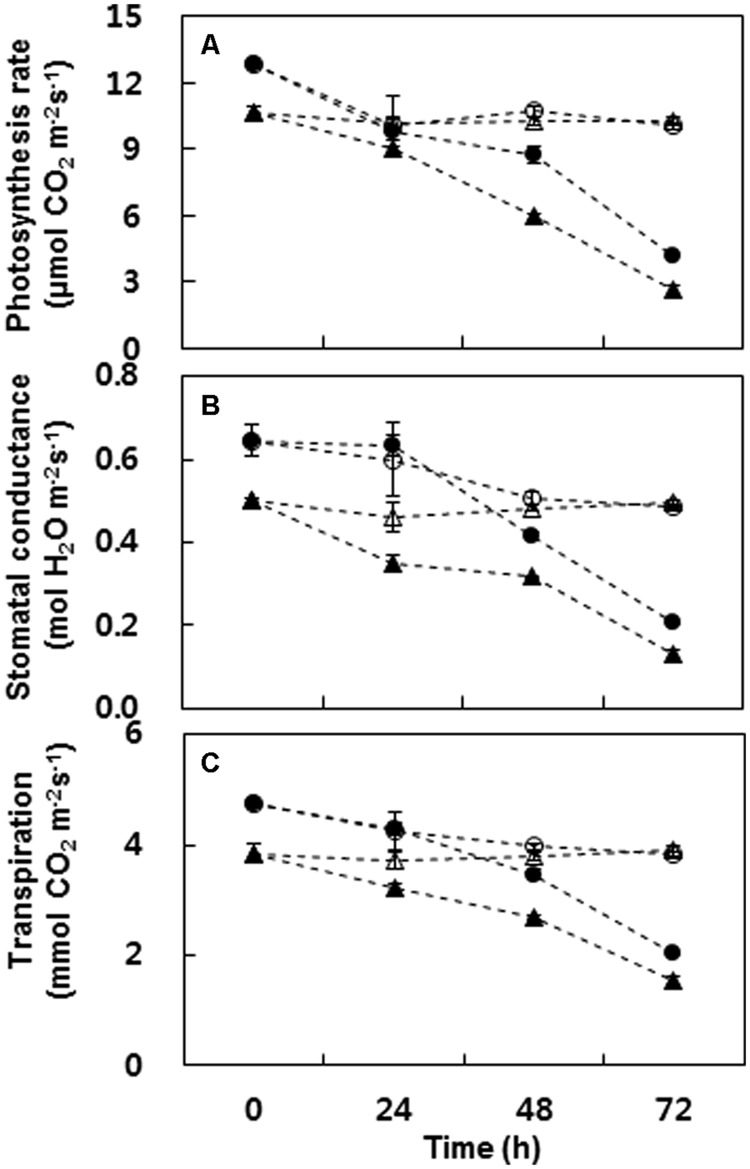
**Changes in photosynthesis rate (A), stomatal conductance (B), and transpiration (C) in cultivars Mosa (triangles) or Saturnin (circles) under control (open symbols) or PEG-induced drought stress (closed symbols) conditions for 72 h.** Data are presented as mean ± SE for *n* = 3. Means denoted by the different letter are significantly different at *P* < 0.05 according to the Duncan’s multiple range test.

Polyethylene glycol-induced drought stress decreased the net photosynthesis rate significantly with varietal difference, representing 74.6 and 58.5% decrease in Mosa and Saturnin, respectively at 72 h after treatment when compared to controls (**Figure 4A**). Varietal difference and drought stress effects on stomatal conductance and transpiration were similar to that of the net photosynthesis rate (**Figures 4B,C**).

## Discussion

The present data allow us to interpret firstly the physiological basis of genetic variation in SUE under PEG-induced drought stress. Secondly, the physiological significance of SUE in drought stress tolerance, with a particular focus on photosynthetic activity, will be discussed for two *B. napus* cultivars with different SUE. Following PEG application for 72 h, drought-stress occurred in both *B. napus* cultivars as shown by Ψ_w_ values (**Table 2**). The final Ψ_w_ in PEG-induced drought stressed plants reached a minimum value of -1.26 and -1.02 MPa, respectively, in Mosa and Saturnin. These values were slightly lower than that (-0.87 MPa) recorded over eight *B. napus* cultivars exposed to water-deficit stress for 7 days (Lee et al., 2015), indicating that PEG provokes drought stress more quickly than water deficiency treatment. These values were, however, higher than that (–1.41 MPa) recorded for four annual clover species exposed to water-stress (Iannucci et al., 2002). The Ψ_w_ has often been used as the criterion for the degree of stress in many studies of stress physiology because the decrease in Ψ_w_, caused by increase in hydraulic or osmotic stress, is responsible for decrease in photosynthetic CO_2_ assimilation rates (Costa Franca et al., 2000), nitrogen assimilation (Kim et al., 2004; Lee et al., 2009), and cell extension processes (Singh et al., 2000).

As expected, PEG-induced drought stress resulted in a reduction of S uptake, estimated by newly absorbed ^34^S amount accumulated during treatment, with varietal difference (**Tables 1** and **3**). The rate of decrease in total amount of S uptake caused by drought stress was generally less in Saturnin (-40.9%) than in Mosa (-63.9%), indicating that Saturnin is able to more efficiently absorb S under drought-stressed conditions. Similarly, Saturnin was estimated to have the highest capacity of N acquisition of eight *B. napus* cultivars exposed to a water deficit stressed condition (Lee et al., 2015). The proportion of NAS distributed to leaves under drought-stressed conditions was also generally higher in Saturnin (76.7% of total S newly absorbed) than in Mosa (58.0%). These results suggest that PEG-induced drought stress restricts the translocation of newly absorbed SO_4_^2-^ to leaves, as a consequence of low SO_4_^2-^ absorption. Low absorption of NO_3_^-^ and decreased translocation into leaves were observed in white clover (Lee et al., 2009) and *B. napus* species with low N use efficiency (Lee et al., 2015) under water deficiency.

Polyethylene glycol-induced drought stress significantly decreased the amount of S assimilated into amino acids (^34^S-amino acids) and proteins (^34^S-proteins). The rate of decrease in ^34^S-proteins was much higher in leaves (-32.8% on average for the two cultivars) than in roots (-28.1%). Similarly, we recently reported that the inhibition of N assimilation into amino acids and proteins due to water-deficit stress occurred more severely in leaves than in roots (Lee et al., 2015). These results indicate that protein synthesis in leaves may be a major sink for S and N assimilation and is likely to be more inhibited by water stress (Kim et al., 2004) and nutrient deficiency (Lee et al., 2013, 2014). Decreases in sulfate uptake, translocation and further assimilation in drought stressed plants (**Table 1**; **Figure 1**) are likely to be driven by low sulfur demand for protein synthesis (De Kok et al., 2002; Anderson and Fitzgerald, 2003). Significant cultivar variation in the impacts of PEG-induced drought stress on *de novo* protein synthesis in leaves was found, with higher rates of decrease of ^34^S-proteins in Mosa (-42.3%) than in Saturnin (-26.8%). A similar finding was made for roots. These results indicate that the inhibition of protein incorporation derived from newly absorbed SO_4_^2-^, caused by drought stress, occurs much less often in cultivars with high S uptake (e.g., Saturnin). This suggests that newly absorbed SO_4_^2-^ is preferentially assimilated into proteins, rather than being stored in vacuoles, so that the capacity of S acquisition is an essential determinant for SUE under drought stress.

For assessing the genotypic variation in tolerance to nutrient deficiency, most studies have focused on nitrogen-use efficiency (Ahmad et al., 2005, 2008; Berry et al., 2010), rather than on SUE. The physiological basis of varietal differences has been mostly interpreted by the relationships between growth and yield characteristics. In this study, cultivar variation in SUE, based on S uptake (SUpE) and S assimilation (SUaE), was assessed using ^34^S tracing. This data, to the best of our knowledge, is the first to directly quantify NAS and its assimilation into amino acids and proteins in response to PEG-induced drought stress. Cultivar variation was significant for SUpE, expressed as mg S taken up per g S, supplied under PEG-induced drought stress treatment (**Figure 2A**). The SUpE of Saturnin was 1.2 times higher in the control and 1.9 times higher in drought stressed plants than those of Mosa. Similarly, SUE based on S assimilation (SUaE), expressed as mg S assimilated per g S supplied, was also significantly higher in Saturnin in both controls and drought stressed plants (**Figure 2B**). In addition, the decreases in SUpE and SUaE caused by PEG-induced drought stress were smaller in Saturnin that those in Mosa. These results indicate that Saturnin is a genotype with a higher SUE compared to Mosa. In a recent study on nitrogen use efficiency based on N uptake (NUpE) and N assimilation (NUaE), Saturnin was also identified as one of the most N-use efficient genotypes of eight *B. napus* cultivars (Lee et al., 2015). This indicates that cultivar variation in SUE is likely to be similar to that of NUE. For *B. napus*, studies carried out under controlled greenhouse (Balint and Rengel, 2008; Dubousset et al., 2010) or field conditions (Schnug et al., 1993; Fismes et al., 2000) have shown that S nutrition affects the N-use efficiency and vice versa.

**FIGURE 5:**
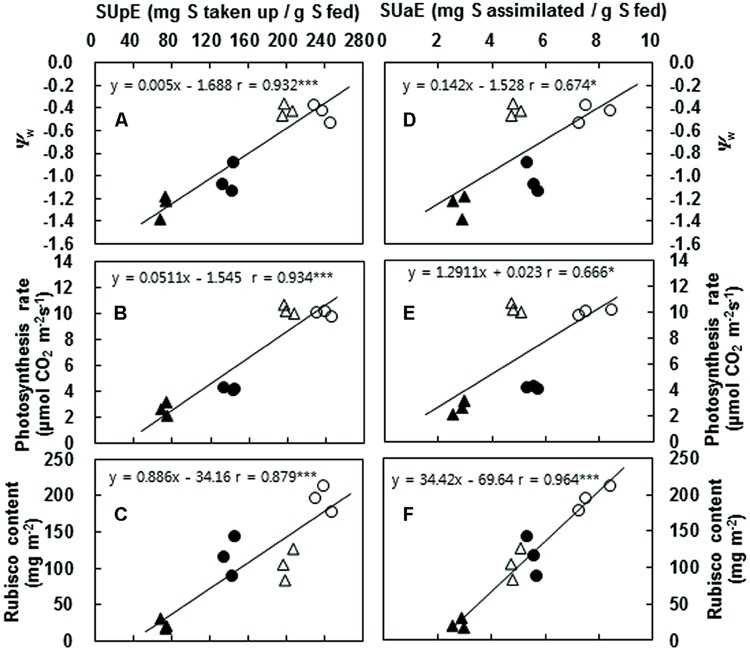
**Correlation of SUpE or SUaE with Ψ_w_ (A,D), photosynthesis (B,E), Rubisco content (C,F) with the values measured at 72 h after control (open symbol) and PEG-induced drought stress (closed symbol) treatment in cultivars Mosa (triangles) or Saturnin (circles).** Significant levels of the linear correlation coefficient were denoted by *P < 0.05, **P < 0.01 and ***P < 0.001.

In this study, PEG-induced drought stress resulted in a gradual decrease in photosynthetic rate, along with a reduction in stomatal conductance and transpiration (**Figure 4**). These results are in accordance with previous results received in experiments with white clover cultivated under water deficiency (Lee et al., 2009). The reduction in the photosynthesis rate results from stomatal closure, which is associated with increased concentrations of ions and other solutes in the cells and the associated decrease in the osmotic potential (Cornic, 1994). Stomatal closure decreases available internal CO_2_ and restricts water loss through transpiration (Chaves, 1991; Tezara et al., 1999). In this study, PEG-induced drought stress degraded Rubisco proteins in both the *B. napus* cultivars examined (**Figure 3**). The inhibition of photosynthetic activity and Rubisco degradation was less significant in Saturnin, which showed a higher efficiency in S uptake and S assimilation (**Figures 3** and **4**). This suggests that a good SUE has the potential to alleviate negative responses caused by drought stress on photosynthetic activity. Indeed, significant correlation of SUpE or SUaE with Ψ_w_ affected by PEG-induced drought stress was found (**Figures 5A,D**). Positive relationships between SUpE or SUaE with net photosynthesis rate and Rubisco content, recorded at 72 h after treatment for the two *B. napus* cultivars examined in this study, were also significant (**Figures 5B,C,E,F**). Reduced photosynthesis under S-deficient conditions has been widely observed in various plant species (Astolfi et al., 2012; Fatma et al., 2014; Muneer et al., 2014). S-deficiency has an early effect on CO_2_ assimilation and on Rubisco activity and protein abundance, thereby inducing chlorosis (Gilbert et al., 1997). The lack of Rubisco synthesis and chlorosis reflects a general inhibition of *de novo* synthesis of photosynthetic apparatus (Hawkesford, 2000). However, in *B. juncea* and *B. campestris*, high S-fertilization increases chlorophyll, Rubisco, and protein contents, and leads to better photosynthetic capacity, compared to the plants grown without S (Ahmad and Abdin, 2000; Fatma et al., 2014). It has also been reported that surplus or sufficient S-supply favors the formation of Fe-S clusters in the photosynthetic apparatus and electron transport system, alleviating the photosynthetic activity inhibited by salt stress (Resurreccion et al., 2002; Nazar et al., 2011) or Fe deficiency (Astolfi et al., 2012; Muneer et al., 2014). Application of exogenous ally isothiocyanate, which is one of the products of hydrolysis of glucosinolates, induced stomatal closure leading to the elevation of guard cell cytosolic Ca^2+^ to avoid water loss (Khokon et al., 2011). Recently it was shown that an excess S supply improved photosynthesis under salt stress condition (Fatma et al., 2014). These positive effects of surplus S-supplementation may be explained by increased S-availability for the formation of S-containing compounds such as amino acids (cysteine and methionine for assembly of new proteins), antioxidant peptides (GSH for protection against oxidative damage), and sulfhydryl (S-H) and disulfide (S-S) bonds that are important for the stabilization of protein structure (Saito, 2000), and S-Fe clusters that function in vital processes such as respiration, photosynthesis, and sulfur and nitrogen metabolism (Balk and Pilon, 2011). These results suggest that SUpE is a determinant for total S-availability, which has significant roles in alleviating negative responses of photosynthetic activity to drought stress, and is a preliminary factor for SUaE. Indeed, the data presented here shows that the inhibition of photosynthetic activity (including Rubisco degradation) is much less apparent in the cultivar with higher SUpE and SUaE (Saturnin), and that this cultivar can be identified as a genotype more tolerant to PEG-induced drought stress, compared to the inefficient SUE cultivar (Mosa).

## Conclusion

This study suggests that SUE based on S uptake (SUpE) and S assimilation (SUaE) displays significant role in alleviating negative responses to drought stress on photosynthetic activity. Thus, an improved SUE is certainly a desired feature for the management of crops against drought stress and for breeding programs with the target to improve the stress tolerance of plants.

## Author Contributions

B-RL and T-HK designed the experiment and wrote the manuscript. B-RL carried out the experiment. T-HK, J-CA, and AO participated in data interpretation and critical reading of the manuscript. RZ has been involved in revising the article for important intellectual content.

## Conflict of Interest Statement

The authors declare that the research was conducted in the absence of any commercial or financial relationships that could be construed as a potential conflict of interest.

## Abbreviations

Ψ_w_: leaf water potential
GSH: Glutathione
NAS: Newly absorbed S
NUaE: N assimilation efficiency
NUpE: N uptake efficiency
PEG: Polyethylene glycol
RSA: Relative specific activities
SUaE: S assimilation efficiency
SUE: Sulfur use efficiency
SUpE: S uptake efficiency
